# Good’s syndrome with opportunistic infection of the central nervous system: a case report

**DOI:** 10.1186/s12883-015-0406-1

**Published:** 2015-08-25

**Authors:** Shinichi Ueno, Satoko Sekimoto-Tsuboi, Yuta Ishiguro, Takahiro Koinuma, Hiroto Eguchi, Yutaka Machida, Nobutaka Hattori, Hideto Miwa

**Affiliations:** Department of Neurology, Juntendo University Nerima Hospital, 3-1-10 Takanodai, Nerima, Tokyo 177-8521 Japan; Department of Neurology, Juntendo University School of Medicine, Tokyo, Japan

**Keywords:** Good’s syndrome, Thymoma, Opportunistic infection, Encephalitis, Cytomegalovirus, Brain

## Abstract

**Background:**

Immunodeficiency with a thymoma (Good’s syndrome) is a rare condition occurring in patients with adult-onset hypogammaglobulinemia that is progressive after the removal of thymoma. Recently, we encountered a patient with Good’s syndrome who suddenly developed opportunistic encephalitis 4 years after the resection of thymoma without a history of infectious complications.

**Case presentation:**

A 58-year-old man, who underwent surgery to remove a thymoma at the age of 54, was admitted because of speech difficulties. A brain MRI showed multiple lesions involving the frontal lobes, but the CSF finding was normal. Acyclovir was empirically administered, and fever as well as his neurological symptoms fully recovered within a few days. However, 1 week after admission, motor aphasia and mild right hemiparesis reappeared. MRI showed that the lesion involving the left cingulate gyrus expanded in size, and revealed an abnormal signal intensity lesion in the left corona radiata. Laboratory examination found increased CMV pp65 antigen-positive lymphocytes in serum. Antiviral therapy using ganciclovir and immunoglobulin replacement therapy was started. The patient has since been free from any neurological symptoms for 1 year, and lesions demonstrated by MRI are gradually improving.

**Conclusion:**

Early recognition of this rare condition and prompt initiation of therapy are crucially important. Awareness of immunodeficiency in a patient after removal of thymoma may help neurologists to consider the possibility that opportunistic infection may be the cause of cerebral lesions.

## Background

It is well known that the thymus has a crucial role in the development of the immune system; however, the detailed mechanisms of its immunological functions remain undetermined. Good’s syndrome is first described as a syndrome of thymoma complicated with hypogammaglobulinemia [1]. Immunodeficiency complicated with thymoma appears in 3–6 % of patients with thymoma [2], and Good’s syndrome is progressive after the removal of thymoma [3]. Recently, we encountered a patient with Good’s syndrome who suddenly developed opportunistic encephalitis 4 years after the resection of thymoma without history of infectious complications.

## Case presentation

**Fig. 1:**
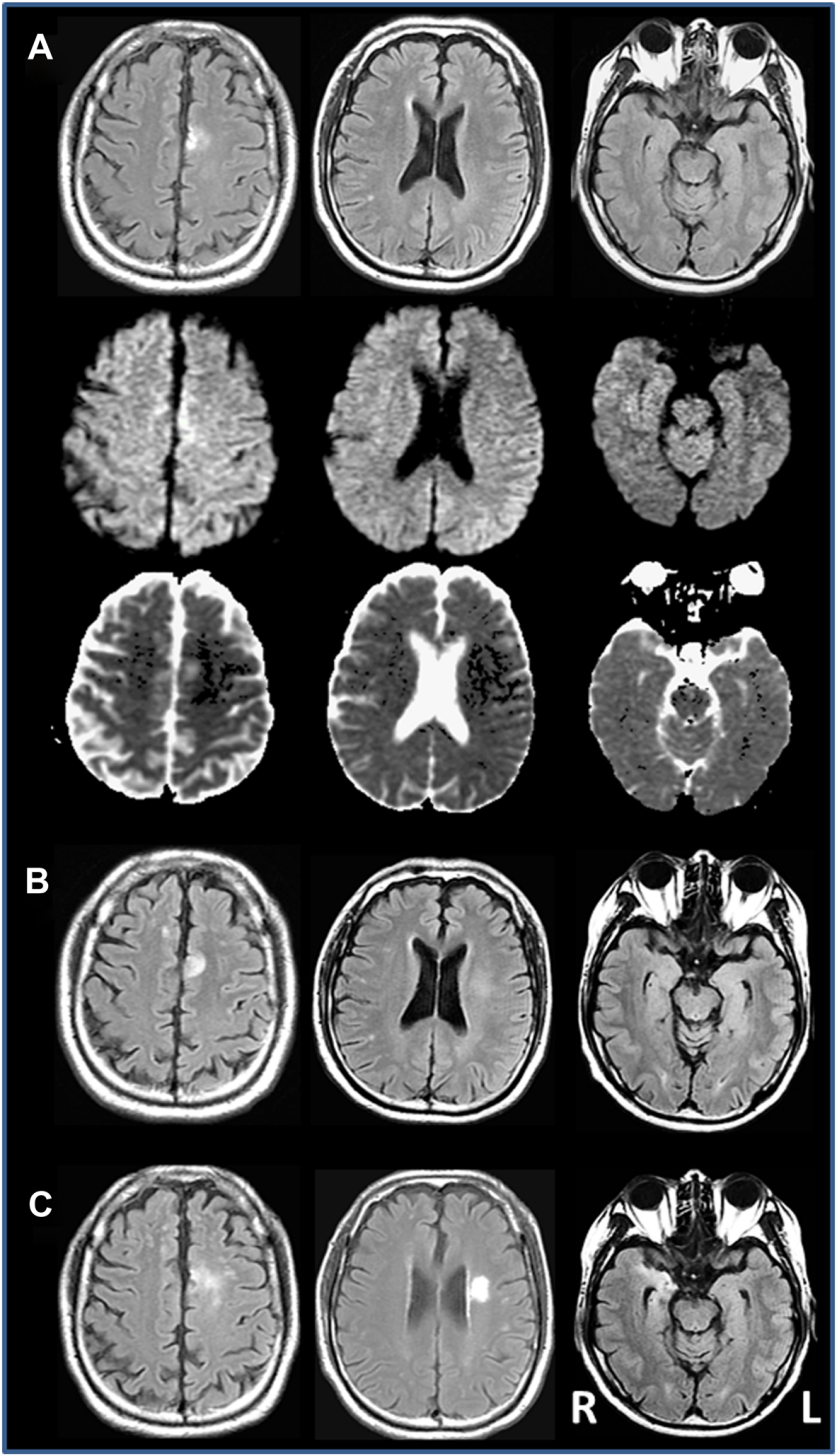
The time course of changes in fluid-attenuated inversion recovery (FLAIR)-image brain MRI of the present patient at admission (**a**, *upper*), 7 days later (**b**), and 10 weeks later (**c**). In panel (**a**), middle and lower are diffusion-weighted imaging (DWI) and ADC maps, respectively. The left cingulate lesion initially expanded in size (**b**), and later became scattered (**c**). The left corona radiate lesion was initially undetectable (**a**), gradually appeared as a vaguely delineated area (**b**), and was later identified as a well-demarcated lesion (**c**). The limbic lesions initially appeared edematous (**a, b**), and later shrunk with a high signal intensity on the right side (**c**)

A 58-year-old Japanese man, who underwent surgery to remove thymoma at the age of 54, was admitted to the emergency room on suspicion of stroke, because he acutely developed speech difficulties. His past medical history was unremarkable except for thymoma that was detected by chance during a health screening. After the thymoma resection, he had been well without recurrence, and received no medical treatment. His family history was also unremarkable. Vital signs were normal except a mild fever (37.8 °C). His general condition was normal (height: 160 cm, weight: 60 kg). Brain MRI demonstrated multiple lesions involving the frontal lobes (Fig. 1a). The left cingulate lesion was partly demonstrated as high-signal intensity in both DWI and ADC maps, suggesting that the lesion contains vasogenic edema. CSF examination was unremarkable, and no elevation of IgG or myelin basic protein was found. EEGs were within normal limits. Because the patient’s neurological findings could not be explained by the cerebral lesions identified in the MRI, we considered the possibility that brain dysfunction might be induced beyond the location of the lesion. Although the CSF findings were normal, acyclovir (10 mg/kg, three times a day) was empirically administered, and his fever and neurological symptoms fully recovered within a few days. However, 1 week after admission, the patient’s symptoms deteriorated again. Neurological examination revealed a reappearance of motor aphasia and mild right hemiparesis. The MRI demonstrated that the lesion involving the left cingulate gyrus increased in size, and an abnormal signal intensity lesion in the left corona radiata, which was presumably the cause of his right hemiparesis, and edematous swelling of the bilateral medial temporal regions appeared (Fig. 1b, c). These lesions were not significantly enhanced by Gadolinium. Although a limbic lesion was demonstrated by MRI, he exhibited no cognitive or psychiatric manifestations. The patient was physically intact without lymphadenopathy. A multi-slice CT scan showed no abnormal findings in his chest and body. CSF was normal. Laboratory studies revealed that the patient’s blood cell counts and chemistry were normal. Of note, marked hypogammaglobulinemia was present, with IgG 481 mg/dL (reference range, 870–1700 mg/dL), IgA 81 mg/dL (reference range, 110–410 mg/dL), and IgM 25 mg/dL (reference range, 33–190 mg/dL). There was a normal CD4/CD8 lymphocyte ratio of 0.70 with CD4 21.9 % and CD8 31.2 %. To take into account the possibility of encephalitis, the patient was screened with tests for infection. Antigens of fungi were negative. Tests for HIV, HBV, HCV, EBV, JC virus, SV40, HHV-6, and HHV-7 were also negative. Particularly, HSV and HZV DNA were repeatedly examined at admission, and 4 and 10 weeks later, and were found to be negative at all time points. CMV DNA in CSF, examined at 10 weeks after admission, was also negative, but CMV pp65 antigen-positive leukocytes were increased in serum (19 antigen-positive cells per 1.5 × 10^5^ cells). Toxoplasma IgG, but not IgM, was elevated in serum. Before and after the thymoma resection, unfortunately, the present patient did not have any immune work-up. However, we believe that his hypogammaglobulinemia was not primary but secondary associated with thymoma, because he had been previously healthy with no susceptibility to infection. Based on his past history of thymoma, hypogammaglobulinemia, and the MRI findings characterized by multiple cerebral lesions involving both gray matter and white matter, the patient was diagnosed as having Good’s syndrome, resulting in opportunistic infections in the brain. To determine the agent responsible for the cerebral lesions, a brain biopsy was recommended; however, it was not performed, according to the patient’s wishes. Because the serum was positive for CMV antigen, antiviral therapy using ganciclovir, 5 mg/kg, twice a day for 2 weeks, was performed. In addition, immunoglobulin replacement therapy (5 g/day, for 3 days) was started and repeatedly performed with an interval of 1 month (5 g/day, every month). The patient has since been free from any neurological symptoms for 1 year, and lesions demonstrated by MRI are gradually improving.

## Conclusions

The present case is characterized by acute neurological symptoms caused by opportunistic infections, which were presumably confined to the brain, caused by immunodeficiency associated with Good’s syndrome. It is clinically important to note that this rare clinical condition can be the cause of opportunistic encephalitis. The pathophysiological mechanisms of Good’s syndrome remain unclear; it remains uncertain why immunodeficiency is associated with thymoma, why immunodeficiency does not improve after the removal of thymoma, and why immunodeficiency is, occasionally, progressively worse even after the removal of thymoma. Indeed, in the present patient, his first symptoms of immunodeficiency appeared 4 years after thymectomy [4].

Opportunistic infections in the CNS are rarely described in Good’s syndrome [5–8]. Generally, recurrent sinopulmonary infections due to encapsulated organisms are common in patients with Good’s syndrome, presumably as a result of the humoral immunodeficiency. In addition, the skin, urinary tract, and gastrointestinal tract are prone to infections [3]. Hematological findings are frequent in routine laboratory examination, and anemia or leukopenia is observed in about half of patients with Good’s syndrome [3, 4]. However, the present case did not show increased susceptibility to such infections, and his hematological data, including blood cell counts and blood chemistry, were normal. Therefore, when we see brain lesions of uncertain cause in patients with a past history of thymoma, the possibility should be considered that the patient may have Good’s syndrome and the lesion may be an opportunistic infection, even if the patient’s lab data are grossly normal.

In Good’s syndrome, several aspects of immunodeficiency may occur in addition to hypogammaglobulinemia, such as low circulating B lymphocytes, CD4+ T-cell lymphopenia, and inverted CD4+ to CD8+ ratio. As a result of cell-mediated immunity defects, patients with Good’s syndrome are likely to develop opportunistic infections, such as CMV, HSV, HZV, candidiasis and Pneumosystis carinii [3, 4]. In the present patient, the infectious agent could not be identified, because a brain biopsy was not performed. However, because the test for CMV was positive, anti-CMV therapy and immunoglobulin replacement therapy were started. Subsequently, the patient’s MRI lesions gradually improved, suggesting that CMV infection, at least partly, may have been the cause of brain lesions in the present patient. However, one diagnostic problem of the present patient is that CMV antigen was positive in leukocytes but CMV DNA was negative in CSF. It thus remains uncertain whether or not the CMV actually invaded the CNS directly. The possibility that an immune-mediated mechanism that was triggered by CMV infection caused the brain damage should also be considered.

Because the prognosis of Good’s syndrome is generally not good [4, 9], early recognition of this rare condition and initiating therapy promptly are crucially important. The present patient did not have any immune work-up after thymectomy, resulting in the initial delay in awareness of Good’s syndrome. Thus, awareness of immunodeficiency in a patient after removal of thymoma may help clinical neurologists to quickly consider the possibility that opportunistic infection may be the cause of cerebral lesions even if the patient does not have episodes suggestive of increased susceptibility to infections.

## Consent

Written informed consent was obtained from the patient for the publication of this case report and accompanying images.

## Abbreviations

CMV: Cytomegalovirus
CSF: Cerebrospinal fluid
EEG: Electroencephalogram
EBV: Epstein-Barr virus
HBV: Hepatitis B virus
HCV: Hepatitis C virus
HHV: Human herpes virus
HIV: Human innmunodeficiency virus
HSV: Herpes simplex virus
HZV: Herpes zoster virus
JCV: JC virus
MRI: Magnetic resonance imaging
SV40: Simian virus 40
